# *Bacillus subtilis* PTA-271 Counteracts Botryosphaeria Dieback in Grapevine, Triggering Immune Responses and Detoxification of Fungal Phytotoxins

**DOI:** 10.3389/fpls.2019.00025

**Published:** 2019-01-24

**Authors:** Patricia Trotel-Aziz, Eliane Abou-Mansour, Barbara Courteaux, Fanja Rabenoelina, Christophe Clément, Florence Fontaine, Aziz Aziz

**Affiliations:** ^1^Research Unit EA 4707 RIBP, SFR Condorcet FR CNRS 3417, University of Reims Champagne-Ardenne, Reims, France; ^2^Department of Plant Biology, University of Fribourg, Fribourg, Switzerland

**Keywords:** grapevine, biocontrol, Botryosphaeria dieback, *Bacillus subtilis*, *Neofusicoccum parvum*, phytotoxins

## Abstract

Plant pathogens have evolved various strategies to enter hosts and cause diseases. Particularly *Neofusicoccum parvum*, a member of Botryosphaeria dieback consortium, can secrete the phytotoxins (-)-terremutin and (*R*)-mellein during grapevine colonization. The contribution of phytotoxins to Botryosphaeria dieback symptoms still remains unknown. Moreover, there are currently no efficient control strategies of this disease, and agro-environmental concerns have raised increasing interest in biocontrol strategies to limit disease spread in vineyards, especially by using some promising beneficial bacteria. Here, we first examined *in planta* the biocontrol capacity of *Bacillus subtilis* PTA-271 against *N. parvum Np*-Bt67 strain producing both (-)-terremutin and (*R*)-mellein. We then focused on the direct effects of PTA-271 on pathogen growth and the fate of pure phytotoxins, and explored the capacity of PTA-271 to induce or prime grapevine immunity upon pathogen infection or phytotoxin exposure. Results provided evidence that PTA-271 significantly protects grapevine cuttings against *N. parvum* and significantly primes the expression of *PR2* (encoding a β-1,3-glucanase) and *NCED2* (9-*cis*-epoxycarotenoid dioxygenase involved in abscisic acid biosynthesis) genes upon pathogen challenge. Using *in vitro* plantlets, we also showed that PTA-271 triggers the expression of salicylic acid- and jasmonic acid-responsive genes, including *GST1* (encoding a glutathione-*S*-transferase) involved in detoxification process. However, in PTA-271-pretreated plantlets, exogenous (-)-terremutin strongly lowered the expression of most of upregulated genes, except *GST1*. Data also indicated that PTA-271 can detoxify both (-)-terremutin and (*R*)-mellein and antagonize *N. parvum* under *in vitro* conditions. Our findings highlight (-)-terremutin and (*R*)-mellein as key aggressive molecules produced by *N. parvum* that may weaken grapevine immunity to promote Botryosphaeria dieback symptoms. However, PTA-271 can efficiently attenuate Botryosphaeria dieback by enhancing some host immune responses and detoxifying both phytotoxins produced by *N. parvum*.

## Introduction

Causal agents of grapevine trunk diseases (GTDs) are very damaging for viticulture since their effect leads to plant death, and to date no grape variety is known to be resistant (Surico et al., 2006; Bertsch et al., 2013; Spagnolo et al., 2014; Fontaine et al., 2015; Magnin-Robert et al., 2016). Botryosphaeria dieback, one of the most threatening GTDs (Bertsch et al., 2013), is caused by several Botryosphaeriaceae fungi, including *Diplodia seriata*, *Diplodia mutila*, and *Neofusicoccum parvum* (Úrbez-Torres, 2011; Larignon et al., 2015). Because of the diversity of these hemibiotrophic fungal pathogens and their virulence characters, understanding the interactions that lead to the disease symptomatology is a major challenge in viticulture. Moreover, the virulence of Botryosphaeriaceae is highly variable within the same species, depending on plant tissue, grapevine cultivar, and environmental conditions (Úrbez-Torres, 2011). A common feature is that Botryosphaeriaceae fungi are mainly found in woody tissues but not in leaves, drawing the hypothesis that secreted fungal toxins delocalized via the xylem sap to the leaves could be involved in the emergence of foliar symptoms (Mugnai et al., 1999). Indeed, several secondary metabolites have been characterized in the Botryosphaeriaceae species (Djoukeng et al., 2009; Evidente et al., 2010; Andolfi et al., 2011; Abou-Mansour et al., 2015), and particular attention has been paid to *Neofusicoccum* spp. regarding its aggressiveness (Úrbez-Torres, 2011). Compounds belonging to two chemical families, the dihydroisocoumarin (*R*)-mellein and the epoxytoluquinol (-)-terremutin as well as their derivatives are considered as the most phytotoxic (Abou-Mansour et al., 2015). Both (*R*)-mellein and (-)-terremutin were detected in wood from vines with Botryosphaeria dieback symptoms (Abou-Mansour et al., 2015), and the produced amounts of (*R*)-mellein were proportional to pathogen aggressiveness (Ramírez-Suero et al., 2014).

(*R*)-Mellein and its derivatives have been isolated not only from pathogens of grapevine, but also from those of apple, pine, citrus and tomato, and are known for their toxicity in different tissues during plant development (Venkatasubbaiah et al., 1991; Parisi et al., 1993; Cabras et al., 2006; Djoukeng et al., 2009; Evidente et al., 2010). It has been shown that (*R*)-mellein induced partial necrosis on grapevine leaves and calli (Djoukeng et al., 2009; Ramírez-Suero et al., 2014; Abou-Mansour et al., 2015), and inhibited the growth of wheat embryo culture (Keller et al., 1994). The (*R*)-mellein derivative methylmellein also exerted a strong antigerminative effect on garden cress (Chooi et al., 2015), while 6-hydroxymellein as a key precursor of (+)-terrein exerted a phytotoxic effect leading to necrotic lesions on fruits (Zaehle et al., 2014; Gressler et al., 2015). (-)-Terremutin and its precursor 6-methylsalicylic acid (6-MSA) as non-host-specific phytotoxins induced necrosis in leaf tissues of grapevine and *Arabidopsis thaliana*, and showed a mild-antibacterial activity (Venkatasubbaiah et al., 1992; Ding et al., 2010). Similarly, the (-)-terremutin derivative terreic acid also showed an antibacterial activity (Yamamoto et al., 1980; Han et al., 2010) and was suspected to be an important antibiotic compound in soil (Chen et al., 2016). In mammals, terreic acid can affect cell’s immunity (Kawakami et al., 1999).

Attention was further paid to the role of fungal toxin systems in the modulation of the plant immune response leading to plant tolerance or susceptibility to pathogens (Pusztahelyi et al., 2015). In this context, (*R*)-mellein and (-)-terremutin were shown to induce a late expression of defense-related genes in grapevine calli, including *Pathogenesis Related* (*PR*) genes and those involved in the detoxification of reactive oxygen species (Ramírez-Suero et al., 2014; Abou-Mansour et al., 2015), but the extent of these responses remained lower compared to those induced by total extracellular pathogen compounds (Ramírez-Suero et al., 2014). More recently, it has been shown that various defense-related genes are not upregulated in grapevine artificially infected with *N. parvum* (Reis et al., 2016; Spagnolo et al., 2017). However, in naturally Botryosphaeria-infected grapevine in vineyards, abundant PR proteins and antioxidant enzymes, as well as stilbene accumulation were reported in the brown striped wood (Spagnolo et al., 2014). Similar trends of gene expression and protein upregulation were observed in grapevine leaves infected with another GTDs, namely Esca-complex (Magnin-Robert et al., 2011; Spagnolo et al., 2012). Interestingly, Magnin-Robert et al. (2016) showed the accumulation of (*R*)-mellein and derivatives in Esca-symptomatic grapevine tissues. However, unlike other pathogens that use specific polyketides as virulence mediators (Uppalapati et al., 2007; Dalmais et al., 2011), to date no relationship was clearly established between (*R*)-mellein or (-)-terremutin accumulation and modulation of the host immune response.

Grapevine like herbaceous or perennial plants can be colonized by an immense number of microbial organisms in the rhizosphere and aboveground parts (Trotel-Aziz et al., 2008; Pinto et al., 2014; Zarraonaindia et al., 2015). Some of these microorganisms can exert either beneficial or detrimental effects (Möbius and Hertweck, 2009; Schroeckh et al., 2009; Pusztahelyi et al., 2015; Zeilinger et al., 2015, 2016). In asymptomatic and symptomatic GTDs-affected grapevines, the bacterial communities also differed in necrotic and non-necrotic tissues. This microbial shift can impact the tolerance or susceptibility of the vine wood to fungal attacks (Bruez et al., 2015). Indeed, some bacteria belonging to *Bacillus* spp. (i.e., *B. subtilis* PTA-271), *Pseudomonas* spp. and *Pantoea* spp. isolated from healthy vineyards, are known to induce systemic resistance against the necrotroph *Botrytis cinerea* (Magnin-Robert et al., 2007; Trotel-Aziz et al., 2008; Verhagen et al., 2011). Beneficial bacteria can directly inhibit pathogen growth and prime plants for enhancing their basal immunity (Verhagen et al., 2004, 2011; Trotel-Aziz et al., 2008; Bakker et al., 2013; Gruau et al., 2015; Aziz et al., 2016). The complex patterns of microbial interactions occurring inside/outside the plant might thus ensure the beneficial outcome of plant association with beneficial/mutualist bacteria in the dieback context. Since 2000, several biocontrol agents have been tested against the numerous pathogens responsible for GTDs, the most efficient to date being antagonistic bacteria and fungi (Haidar et al., 2016; Mondello et al., 2018). For instance, *Trichoderma* spp. generally showed high efficiency in wound protection against all GTDs pathogens (Di Marco et al., 2002, 2004; John et al., 2008; Halleen et al., 2010) as well as *Bacillus* spp. (Schmidt et al., 2001; Halleen et al., 2010; Kotze et al., 2011; Rezgui et al., 2016). The benomyl-resistant mutant *Fusarium lateritium* strain was especially effective as a wound protectant against *Eutypa lata* (McMahan et al., 2001; John et al., 2005). This strain can degrade *in vitro* some phytotoxins involved in the expression of foliar symptoms, namely eutypine, 4-hydroxybenzaldehyde, and 3-phenyllactic acid produced by *E. lata* and pathogens from Esca consortium (Christen et al., 2005). In contrast, the rhizospheric *Pythium oligandrum* was shown to reduce *Phaeomoniella chlamydospora* wood necrosis (Esca complex) by stimulating host plant defenses (Benhamou et al., 2012; Yacoub et al., 2016).

Although several biocontrol agents were successfully tested against GTDs pathogens (Mondello et al., 2018), few studies tried to decipher mechanisms involved in plant protection against Botryosphaeria species and their aggressive molecules. Especially, the molecular mechanisms underlying induced protection, and the extent by which beneficial bacteria modulate grapevine immunity and detoxification of the virulent-phytotoxins (*R*)-mellein and (-)-terremutin, remain largely unknown. In this study, we first examined the capacity of the beneficial bacterium *B. subtilis* PTA-271 (hereafter PTA-271) to counteract grapevine infection by a *N. parvum* strain producing both (-)-terremutin and (*R*)-mellein (namely *N. parvum*-Bt67). We then focused on the effects of PTA-271 on pathogen’s growth and removal of pure phytotoxins from growth medium. We finally explored the capacity of PTA-271, which was initially isolated from grapevine rhizosphere, to induce or prime grapevine immunity upon pathogen inoculation or after plant exposure to exogenous phytotoxins.

## Materials and Methods

### Plant Material and Growth Conditions

Three-node-long cuttings of grapevine (*Vitis vinifera* L., cv. Chardonnay) were collected from 10-year-old plants in Pommery’s vineyards in Reims (France) and kept in a cold chamber at 4°C for 1 month. Cuttings were surface-sterilized with 0.05% cryptonol (8-hydroxyquinoline sulfate) and rooted as described by Lebon et al. (2005). They were placed in 350 mL pots containing the soil Gramoflor Special (Gramoflor GmbH & Co. KG, Vechta, Germany) in a culture chamber (25°C day/night, 60% relative humidity, and 16 h photoperiod at 400 μmoles/m^2^/s) and watered twice a week. Only cuttings that have developed roots were conserved for further experiments.

Grapevine plantlets (*V. vinifera* L. cv. Chardonnay, clone 7535) were produced from nodal explants transferred on 15 mL of agar-modified Murashige-Skoog (MS) medium (Trotel-Aziz et al., 2008) in 25-mm test tubes. Plantlets were grown at 25°C day/night, with a 16/8 h photoperiod.

### Bacterial Growth and Treatment

*Bacillus subtilis* PTA-271 (GenBank Nucleotide Accession No. AM293677) was isolated from the rhizosphere of healthy field-grown Chardonnay grapevines in Champagne area, France (Trotel-Aziz et al., 2008). Bacterial growth starts by adding 100 μl of the glycerol stock suspension to sterile Luria Bertani (LB) medium, before incubating at 28°C under continuous shaking (75 rpm). Experiments were performed with the bacteria at the exponential growth phase. After centrifugation (5000 *g*, 10 min), the pellet was washed once and resuspended in sterile 10 mM MgSO_4_ medium. Bacterial density was measured by spectrophotometry at 450 and 650 nm, and the mean concentration was adjusted with sterile MgSO_4_ medium before treatment.

Bacterial suspension was applied twice at the root level of cuttings at a final concentration of 10^8^ cfu/g soil. The first inoculation was performed when cuttings were 8 weeks old and the second inoculation when cuttings were 10 weeks old. Control cuttings were thus similarly drenched twice with MgSO_4_ solution.

For *in vitro*-plantlets, bacterial suspension was adjusted to 10^8^ cfu/mL with sterile liquid MS medium then added in new sterile 25-mm culture-tubes (15 mL per tube). Six-week-old plantlets were then transferred in these new tubes for 2 weeks of bacterial treatment in a growth chamber at 22°C with a photoperiod 16/8 h. Control plantlets were transferred in liquid MS medium without bacteria under the same conditions.

### Fungal Strain and Growth

The *N. parvum* strain *Np*-Bt67 (Reis et al., 2016) isolated from Portuguese vineyards (Estremadura area) is inscribed in HIA collection (Lisbon University, Portugal). Fungi was maintained on potato dextrose agar (PDA, Sigma, Saint-Quentin-Fallavier, France) plates and stored at 4°C. Resulting mycelium was plated on PDA medium and incubated in the dark at 22°C for 7 days before used to inoculate cuttings.

### Production and Quantification of Phytotoxins

(*R*)-mellein (log Kow ∼ 2.5) and (-)-terremutin (log Kow ∼ 0) were extracted and purified from a 10-day-old culture of the *Np* strain, according to Abou-Mansour et al. (2015). Both toxins were prepared as concentrated stock solutions in sterile MS or 10 mM MgSO_4_ medium and stored in the dark at 4°C. Before each experiment, daughter solutions were prepared for the biological experiments, and the phytotoxin concentrations were determined before and after treatment using HPLC coupled to a diode array detector (Ultimate 3000 Dual-Gradient, Dionex, Voisins-le-Bretonneux, France). Analyses were done on a C18 reversed phase column (100 mm× 3 mm, 5 μm, Kromasil 100, Dionex) using isocratic elution with acetonitrile (ACN, LC–MS quality, Merck, France) and water (H_2_0) containing 0.1% phosphoric acid (H_3_PO_4_). Detection was recorded at 210 and 273 nm for (*R*)-mellein and (-)-terremutin, respectively. Phytotoxin identification was confirmed by UV spectrum and retention time; (*R*)-mellein was eluted with 1 mL/min of ACN:H_2_O 60:40 v/v at 3.8 min, while (-)-terremutin was eluted with 0.7 mL/min of ACN:H_2_O 10:90 v/v at 5.8 min. Concentration was determined using standard curves.

### Fungal Inoculation and Disease Expression

Cuttings pretreated 1 month with bacteria were then wounded (5 mm diameter, 1 mm deep) at 12 weeks old at the second node of the green stem and inoculated with a 3 mm diameter mycelial plug from the 7-day-old culture of *Np-*Bt67 strain. Inoculation site was then covered with moisten hydrophilic cotton before sealing with parafilm. Without bacteria, cuttings were pretreated 1 month with MgSO_4_, then pathogen-inoculated also at 12 weeks old using the same method. To confirm that lesions were really due to pathogen infection and not to the injury, controls were inoculated with sterile 3-mm PDA plugs. After inoculation, cuttings were kept in the same culture chamber conditions to quantify Botryosphaeria dieback symptoms at 4 months post-inoculation. As potentially indicative, phytotoxins were also extracted from the same leaf powder (1 g FW in 5 mL of methanol – LC–MS quality, Merck, France *–* for 1 h at 37°C before analysis in supernatant as described below) at least twice in triplicates, and phytotoxins were not detectable in leaves of infected cuttings. At 4 months post-inoculation, symptoms of Botryosphaeria dieback were evaluated by measuring both the canker and necrotic surface area on green shoots as described by Espinosa et al. (2009) and Laveau et al. (2009), and by quantifying the percentage of dead branch for inoculated cuttings.

### Evaluation of Direct Effect of *B. subtilis* PTA-271 on *N. parvum* Growth

PTA-271 grown in LB medium was inoculated (5 μL drop at 10^9^ cfu/mL) on the one side of a Petri plate (9 cm diameter) containing PDA medium, then incubated at 28°C in the dark. After 24 h, a mycelium plug of 4-day-old pathogenic fungus was co-inoculated on the other side of PDA plates, and the plates were incubated in the same conditions. Controls are PDA plates with a mycelium plug and a LB-drop incubated until mycelial growth reached the edge of the control plate. The same experiment was also performed at 22°C as an optimal temperature for pathogen growth (Trotel-Aziz et al., 2008), while 28°C was optimal for PTA-271 growth. Antagonistic effect was characterized by an inhibition zone around bacterial colony.

### Detoxification Assays With *B. subtilis* PTA-271

PTA-271 was collected at exponential phase in LB medium, diluted to reach a final density of 10^4^ to 2 × 10^8^ cfu/mL, and centrifuged at 5000 *g* (4°C, 15 min). Pellet was then resuspended either in a sterile MS medium (nutrient rich) or in a 10 mM MgSO_4_ medium (nutrient poor) containing or not (*R*)-mellein 350 μg/L ( = 100%) or (-)-terremutin 750 μg/L ( = 100%). Detoxification tests were performed after assessing the toxicity of (*R*)-mellein and (-)-terremutin on both bacteria and plantlets (see data in Supplementary Figures S1, S2). For both molecules, no toxic effect was observed from 0 to 1500 μg/L neither on the plant nor on the bacterium. Detoxification assays were done in triplicate at 28°C under continuous shaking for 72 h. Percentage of each phytotoxin was determined daily in both bacterial pellet and supernatant (culture medium) obtained after centrifugation. Phytotoxins were extracted from bacterial pellet with acetone (HPLC quality, VWR, France) by shaking for 48 h in darkness at 4°C. Mixture was then centrifuged (5000 *g*, 15 min, 4°C) and clean supernatant was collected for direct phytotoxin analysis with HPLC as described before. (-)-Terremutin as a highly hydrophilic molecule was directly analyzed in the culture medium by direct injection into HPLC system. However, (*R*)-mellein was extracted from the culture medium with hexane (10:2 v/v, extraction yield > 90%). After a vigorous shake of 1 min, the upper organic phase was directly used for (*R*)-mellein analysis with HPLC. Two controls were carried out: living bacteria in a toxin-free medium as a biological control, and medium containing only toxin without living bacteria as a physicochemical control.

### Treatment of Grapevine Plantlets With (*R*)-Mellein and (-)-Terremutin

To investigate phytotoxin’s capacity to modulate plant immunity, 6 weeks old plantlets were treated with bacterial suspension in liquid MS medium at the root level. After 2 weeks, roots of were washed three times in sterile liquid MS, then plantlets were transferred in a new sterile liquid MS medium supplemented or not with (*R*)-mellein 350 μg/L or (-)-terremutin 750 μg/L for 72 h under growth chamber conditions. Controls consisted of 8 weeks old plantlets on MS medium, further transferred for 3 days on liquid MS medium with or without phytotoxins.

In the meantime, phytotoxins were quantified from plantlet’s incubating medium as described before, and extracted from shoot and roots with methanol (weight/volume: 1/5) in darkness under continuous shaking for 48 h at 4°C. The homogenate was then centrifuged at 5000 *g* for 15 min at 4°C and the clean supernatant was directly used for phytotoxin analysis by HPLC. All experiments were repeated four times at least in triplicate. Two different controls were carried out: living plants in a toxin-free medium and medium containing only toxin without living plants.

### RNA Extraction and qRT-PCR Analysis

Leaf samples from cuttings and shoots from plantlets were collected respectively at 4 days post-inoculation with pathogen and at 3 days post-treatment with phytotoxins, ground in liquid nitrogen then stored at -80°C. Total RNA were extracted from 50 mg of leaf powder for cuttings or from 100 mg of powdered plantlet shoots with PlantRNA Purification Reagent according to manufacturer instructions (Invitrogen, Pontoise, France), and DNase treated as described by Gruau et al. (2015). RNA quality was checked by agarose gel electrophoresis, and total RNA concentration was measured at 260 nm for each sample and adjusted to 100 ng μL^-1^. First-strand cDNA was synthesized from 150 ng of total RNA using the Verso cDNA synthesis kit (Thermo Fisher Scientific, Inc., Waltham, MA, United States). PCR conditions were those described by Gruau et al. (2015). Quantitative RT-PCR was performed with Absolute Blue qPCR SYBR Green ROX Mix according to manufacturer instructions (Thermo Fisher Scientific, Inc., Waltham, MA, United States), in a BioRad C1000 thermocycler using the BioRad manager software CFX96 Real Time PCR (BioRad, Hercules, CA, United States). A set of 13 defense-related genes, selected for their responsiveness to pathogen or priming state induced by beneficial bacteria (Spagnolo et al., 2012, 2014; Gruau et al., 2015; Magnin-Robert et al., 2016), was tracked by quantitative reverse-transcription-polymerase chain reaction (qRT-PCR) using specific primers (Supplementary Table S1). qRT-PCR reactions were carried out in duplicates in 96-well plates in a 20-μl final volume containing Absolute Blue SYBR Green ROX mix including Taq polymerase ThermoPrime, dNTPs, buffer and MgCl_2_ (Thermo Fisher Scientific, Inc., Waltham, MA, United States), 280 nM forward and reverse primers, and 10-fold diluted cDNA according to the manufacture’s protocol. Cycling parameters were 15 min of Taq polymerase activation at 95°C, followed by 40 two-step cycles composed of 10 s of denaturation at 95°C and 45 s of annealing and elongation at 60°C. Melting curve assays were performed from 65 to 95°C at 0.5°C⋅s^-1^, and melting peaks were visualized to check amplification specificity. *EF1* and *60SRP* genes were used as references and experiments were repeated five times. Relative gene expression was determined with the formula fold induction: 2^(-ΔΔ*Ct*)^, where ΔΔCt = [Ct TG (US) – Ct RG (US)] – [Ct TG (RS) – Ct RG (RS)], where Ct is cycle threshold, Ct value is based on the threshold crossing point of individual fluorescence traces of each sample, TG is target gene, RG is reference gene, US is unknown sample, and RS is reference sample. Integration of the formula was performed by the CFX Manager 3.0 software (BioRad). The genes analyzed were considered significantly up- or down-regulated when changes in their expression were > 2-fold or < 0.5-fold, respectively. Control samples for the cuttings model are cDNA from leaves of cuttings untreated with bacteria and inoculated with sterile PDA plugs (1x expression level), while for the *in vitro* model it corresponds to shoots from plantlets grown on MS medium without PTA-271 and phytotoxins (1x expression level).

### Statistical Analysis

To quantify phytotoxins, standard curves were first established with pure phytotoxins through titrations repeated at least three times from two independent experiments. Biocontrol assays with cuttings model were repeated at least three times with at least 10 cuttings per treatment. The confrontation tests between PTA-271 and fungal pathogen were triplicated in experiments conducted twice. Detoxification assays with *in vitro* PTA-271 or *in vitro* plantlet model were repeated four times with each sample at least triplicated. Data are means ± standard deviations. Analyses of gene expression by qRT-PCR were repeated five-times from independent experiments. RNAs were extracted from powdered 20 leaves of 10 grapevine cuttings, and from powdered shoots of four plantlets. Results correspond to means ± standard deviation from one representative out of at least three showing the same trends. Statistical analyses were carried out using the SigmaStat 3.5 software. For treatment effect, mean values were compared by Tukey’s test (*P* < 0.05).

## Results

### *B. subtilis* PTA-271 Attenuates Botryosphaeria Dieback Symptoms in Grapevine Cuttings

**FIGURE 1:**
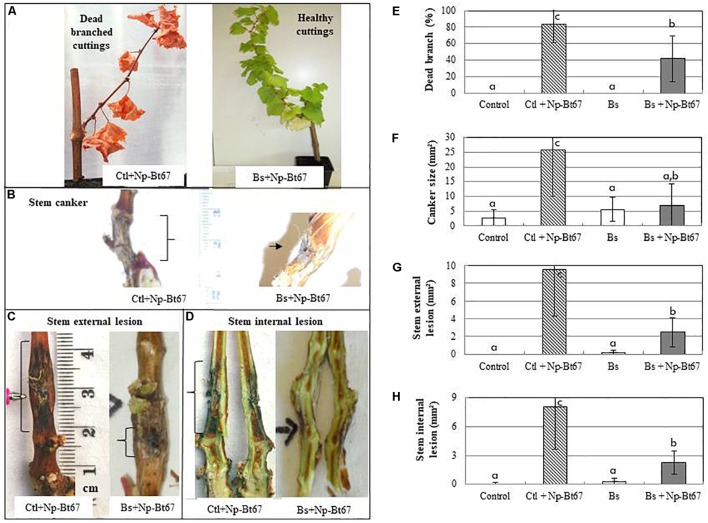
The beneficial bacterium *Bacillus subtilis* PTA-271 attenuates the characteristic Botryosphaeria dieback symptoms induced in Chardonnay cuttings by the *Neofusicoccum parvum* strain *Np*-Bt67. One month pretreated grapevine cuttings with PTA-271 (Bs, 2 × 10^8^ cfu/g soil) and non-bacteria pretreated ones (Ctl) were inoculated with pathogen mycelium (+Np-Bt67). Non-infected plants were inoculated with sterile medium without pathogen (Control). Compared to PTA-271 treated healthy asymptomatic cuttings **(A)**, the infected symptomatic cuttings showed the typical Botryosphaeria dieback symptoms: dead branch **(A,E)**, stem canker **(B,F)**, stem internal necrosis **(C,G)**, and stem external necrosis **(D,H)** that were photographed **(A–D)** and quantified **(E–H)** at 4 months post-inoculation. Data are means ± standard deviation (SD) for at least three independent experiments with 10 biological replicates per treatment. Vertical bars with different letters are significantly different (Multiple Comparison procedures with Tukey’s test, *P* < 0.05).

PTA-271 was used to evaluate its capacity to control the occurrence of Botryosphaeria dieback symptoms. Bioassays with Chardonnay cuttings from control or bacteria-pretreated plants at root level further inoculated with *Np*-Bt67 showed that PTA-271 significantly reduced the dead branch development (Figure 1A) by approximately 50% compared to non-bacteria pretreated plants (Figure 1E). Similarly, the size of canker (Figure 1B) and those of external and internal stem lesions (Figures 1C,D) were reduced in PTA-271-pretreated cuttings after challenge with *Np*-Bt67. Both canker and stem lesions were reduced by about 63 to 75% compared to non-bacteria pretreated plants (Figures 1F–H). These results indicate that PTA-271 could efficiently protect Chardonnay cuttings from the *N. parvum* strain *Np*-Bt67.

### *B. subtilis* PTA-271 Antagonizes *N. parvum* and Detoxifies Both (*R*)-Mellein and (-)-Terremutin

**FIGURE 2:**
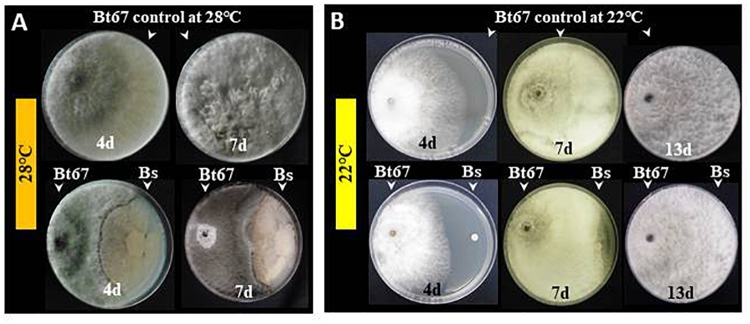
Antagonistic activity of *B. subtilis* PTA-271 toward the *N. parvum* strain *Np*-Bt67. The beneficial bacterium PTA-271 (Bs) and the *N. parvum* strain *Np*-Bt67 (Bt67), co-inoculated on the opposite sides of PDA plates, were incubated at 28°C **(A)** or 22°C **(B)**. Pictures of representative plates among nine were taken from 4 to 13 days depending on mycelial growth. Top photographs are the plates without bacteria (pathogen control) and bottom ones are the plates co-inoculated with pathogen and Bs. Antagonism effect is characterized by an inhibition zone between the bacterial colony (right side) and the fungus (left side).

In regard to *in vitro* test with pathogen mycelium, results showed that PTA-271 clearly antagonizes *Np*-Bt67 by a fungistatic effect compared to control treatment at 28°C (Figure 2A) in both time points. Antifungal effect was detected approximately 4 days after pathogen inoculation at 22°C (Figure 2B). Thereafter, mycelial growth increased progressively and became comparable to the control.

**FIGURE 3:**
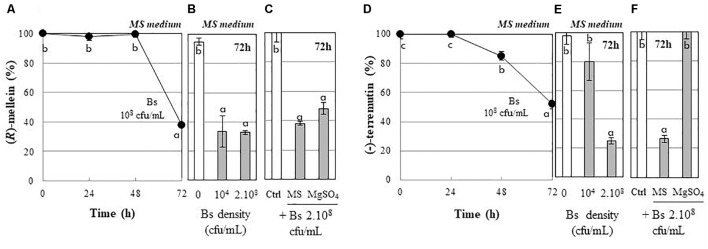
Detoxifying capacity of *B. subtilis* PTA-271 (Bs) toward the purified (*R*)-mellein **(A–C)** and (-)-terremutin **(D–F)** from *N. parvum*. Phytotoxin concentrations were determined as remaining percentages in the bacterial incubating media either: **(A,D)** daily form MS medium containing PTA-271 (Bs) at 10^8^ cfu/mL, or **(B,E)** 72 h post-exposure to two distinct bacterial densities 10^4^ and 2 × 10^8^ cfu/mL in MS, or **(C,F)** 72 h post-exposure to the two distinct incubating media Murashige-Skoog medium (MS) and MgSO_4_ with *Bs* at 2.10^8^ cfu/mL. Data are means ± SD of three independent experiments, each with triplicates. The toxin controls (Ctrl) indicated none physicochemical disappearance. Phytotoxins were not detectable inside bacterial pellet. Vertical bars with different letters are significantly different (Multiple Comparison procedures with Tukey’s test, *P* < 0.05).

We also investigated whether PTA-271 can affect fungal toxins, (*R*)-mellein and (-)-terremutin exogenously applied to their culture medium. Results showed that the percentage of both (*R*)-mellein and (-)-terremutin was significantly decreased in the presence of PTA-271 (Figure 3). The (*R*)-mellein decrease was effective after a 48 h latency period in the presence of PTA-271 at 10^8^ cfu/ml, and reached 40% after 72 h of exposure (Figure 3A). Similar effect was observed after 72 h of incubation with PTA-271 at low (10^4^ cfu/ml) or high (2 × 10^8^ cfu/ml) bacterial density (Figure 3B). In addition, the bacterium seems to be effective to remove (*R*)-mellein, whether suspended in MS medium or in the less nutrient rich MgSO_4_ medium. The amount of (*R*)-mellein decreased significantly with the high bacterial density whether in MS medium or in the less nutrient rich MgSO_4_ medium (Figure 3C). Interestingly, the (-)-terremutin decrease was effective after a 24 h latency period in MS medium in the presence of PTA-271 at 10^8^ cfu/ml to reach 50% after 72 h (Figure 3D). Such a (-)-terremutin decrease was not observed in the presence of the bacterium at lower density (Figure 3E), or in the less nutrient rich MgSO_4_ medium compared to MS one (Figure 3F).

### *B. subtilis* PTA-271 Strongly Primes the Expression of *a β-1,3-Glucanase* After *N. parvum* Inoculation in Grapevine Cuttings

**FIGURE 4:**
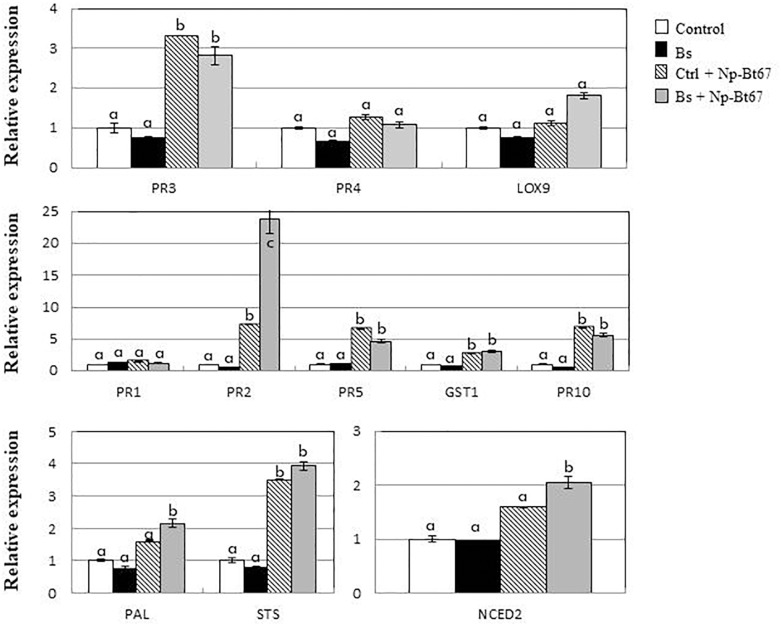
*B. subtilis* PTA-271 strongly primes *PR2* gene in leaves of grapevine cuttings after infection with the *N. parvum* strain *Np*-Bt67. Twelve weeks old plantlets untreated or pretreated with PTA-271 were both infected with sterile PDA plugs (Control and Bs, respectively) or with mycelium plugs of *Np*-Bt67 (Ctl + Np-Bt67 and Bs + Np-Bt67, respectively). Transcript levels of defense-related genes were monitored by qRT-PCR in leaves at 4 days post infection. Results are from one representative replicate among three independent experiments showing the same trends. Different letters indicate significant differences. *PR3* = class IV chitinase (*chit4c*); *PR4* = PR-4 type protein; *LOX9* = lipoxygenase 9; *PR1* = pathogenesis-related protein 1; *PR2* = class I β-1,3-glucanase; *PR5* = thaumatin-like protein; *GST1* = glutathione-S-transferase 1; *PR10* = pathogenesis-related protein 10; *PAL* = phenylalanine ammonia lyase; *STS* = stilbene synthase; *NCED2* = 9-cis-epoxycarotenoid dioxygenase 2.

In leaves of control cuttings inoculated with *Np*-Bt67, data from qRT-PCR (Figure 4 and Supplementary Figure S3) showed that, except for *PR1* (1.4-fold expression), the expression of defense genes responsive to salicylic acid (SA) including *PR2*, *PR5*, and *PR10* was significantly up-regulated from 6.6- to 7.3-fold. Expression of *PAL* (phenylalanine ammonia-lyase) and *STS* (stilbene synthase) involved in the synthesis of phytoalexins was also increased by 1.6- and 3.5-fold, respectively. In the meantime, expression of *GST1* encoding a glutathione-*S*-transferase putatively involved in the detoxification process, and that of *PR3* and *PR4* as responsive to jasmonic acid/ethylene (JA/ET), was upregulated by 2.7-, 3.3- and 1.3-fold, respectively. Data also showed a low upregulation of the *NCED2* gene involved in abscisic acid biosynthesis (1.6-fold), while that of *LOX9* was not upregulated by *N. parvum*.

The ability of PTA-271 to enhance grapevine immunity was addressed. Gene expression levels after pretreatment with PTA-271 was similar to control plants, before pathogen challenge (Figure 4 and Supplementary Figure S3). However, after *Np*-Bt67 inoculation, bacteria-pretreated plants showed a significant priming of *PR2* (encoding a β-1,3-glucanase), *NCED2 and PAL* expressions compared to non-bacteria pretreated plants. *PR2* mRNA level was more markedly primed in the leaves (23.7-fold). However, only slight differences were observed regarding transcript levels of *LOX9*, *GST1*, and *STS*, while the expression levels of *PR1*, *PR3*, *PR4*, and *PR10* did not change in bacteria-treated plants compared to control after pathogen infection.

### *N. parvum* Phytotoxins Repress PTA-271-Mediated SA- and JA-Responsive Gene Expression in Grapevine Plantlets

**FIGURE 5:**
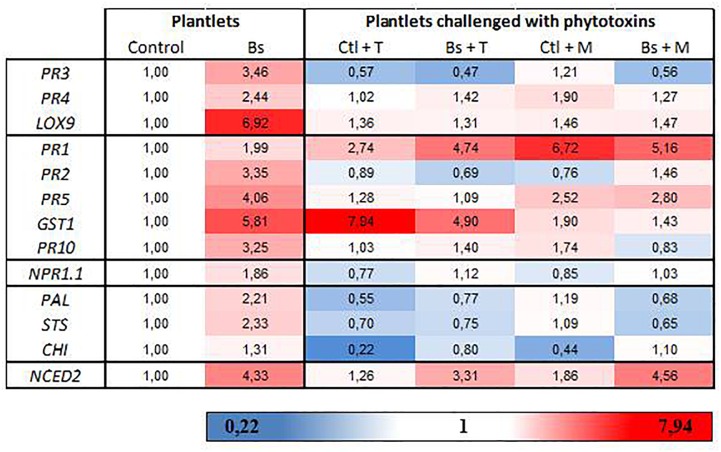
(*R*)-mellein and (-)-terremutin repress the *B. subtilis*-PTA-271-induced immune responses in grapevine plantlets. Eight weeks old plantlets untreated or pretreated with PTA-271 were further challenged with MS medium (Ctl and Bs, respectively) supplemented with (-)-terremutin (Ctl+T and Bs+T, respectively) or (*R*)-mellein (Ctl+M and Bs+M, respectively). Transcript levels of defense-related genes were monitored by qRT-PCR in plantlets shoots after 3 days of exposure. Results are from one representative replicate among five independent experiments showing the same trends. A three-color scale was used to show the expression level of each gene. Red shades indicate overexpression and deep red corresponds to an induction factor of 7.94 or more; white represents the basal expression level and signifies that the expression level is not different from the Control; blue shades symbolize repression and dark blue corresponds to a 0.22-fold induction or less. Legends for genes are as in Figure 4. *CHI* = chalcone isomerase; *NPR1.1* = non-expresser of *PR* genes 1.

To focus on the repression of gene expression induced by PTA-271 after toxin application, we first examined how the bacterium affects gene expression in plantlets leaves. Data (Figure 5 and Supplementary Figure S4) showed that PTA-271 alone induced significant changes in the expression of genes responsive to JA/ET (*PR3*, *PR4*, *LOX9*), SA (*PR1*, *PR2*, *PR5*, *GST1*, *PR10*) or abscisic acid ABA (*NCED2*, involved in ABA synthesis) compared to control plantlets. Transcript level was increased from 2.4- to 6.9-fold for JA/ET-responsive genes, from 2.0- to 5.8-fold for SA-responsive ones, and by 4.3 for *NCED2*. Expression of *PAL* and *STS* was also increased by 2.2 and 2.3-fold, respectively, and to a lesser extent for *CHI* (chalcone isomerase) and *NPR1.1* (non-expresser of *PR1*) reaching 1.3- and 1.9-fold expression, respectively.

After a subsequent exposure to toxins, most of the defense genes induced by PTA-271 were repressed. (-)-Terremutin and (*R*)-mellein significantly repressed the expression of genes responsive to JA/ET (*PR3*, *PR4*, *LOX9*) and SA (*PR2*, *PR5*, *PR10*), and that of PAL and STS (involved in phenylpropanoid pathway). (*R*)-mellein additionally repressed the expression *GST1*, another gene responsive to SA. Expression of the SA-dependent *PR1* gene was the sole gene still over induced in PTA-271 treated plantlets after toxin application, as in control plantlets treated with both toxins. Expression of another SA-dependent *GST1* gene was the sole gene still over induced in PTA-271 treated plantlets after (-)-terremutin application, as in control plantlets treated with (-)-terremutin. In contrast, expression of the ABA-dependent *NCED2* gene was the sole gene still over induced in PTA-271 treated plantlets after each toxin application, while not significantly in control plantlets treated with toxins.

### (*R*)-Mellein and (-)-Terremutin Are Mobilized or Accumulated Differently by PTA-271-Pretreated Plantlets

**FIGURE 6:**
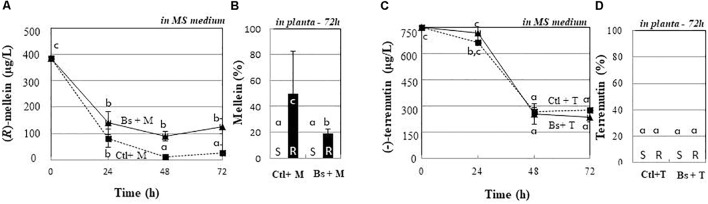
Fate of (*R*)-mellein and (-)-terremutin from plantlets incubating medium or from medium of *B. subtilis*-PTA-271-pretreated plantlets. Eight weeks old plantlets treated with PTA-271 were transferred in a new MS medium containing (*R*)-mellein (Bs + M) 350 μg/L **(A,B)** or (-)-terremutin (Bs + T) 750 μg/L **(C,D)**. The same experiment was performed with non-bacteria pretreated plantlets, then transferred on (-)-terremutin 750 μg/L (Ctl + T) or (*R*)-mellein 350 μg/L (Ctl + M). Phytotoxin concentrations were determined either: daily in the plant culture media from 0 to 72 h **(A,C)**, or 72 h post-exposure as percentage accumulated in shoots (S) and roots (R) **(B,D)**. Data are means ± SD of three independent experiments, each with triplicates (at least four plantlets by replicate). The toxin controls indicated none physicochemical disappearance. Phytotoxins were not detectable inside bacterial pellet. Vertical bars with different letters are significantly different (Multiple Comparison procedures with Tukey’s test, *P* < 0.05).

To investigate the fate of phytotoxins in the incubating medium of plantlets, control and PTA-271-pretreated plants were exposed to (*R*)-mellein or (-)-terremutin at their root level. As shown in Figure 6A, (*R*)-mellein quickly decreased in the MS growth medium of control plantlets. The amount of (*R*)-mellein decreased by about 83.5% within 24 h and by 97.5% after 48 h. *In planta* (Figure 6B), roots accumulated about 50% of (*R*)-mellein within 72 h. Experiments with PTA-271-pretreated plantlets showed a partial (*R*)-mellein removal even after 72 h exposure (Figure 6A), thus confining 20% of (*R*)-mellein in the incubating medium (Figure 6A), while only 20% of (*R*)-mellein was accumulated inside the plantlet roots (Figure 6B).

Supplied (-)-terremutin also decreased significantly from plantlets incubating medium (Figure 6C), especially after a 24 h period of exposure, to reach about 35% from 48 h. At 72 h, no apparent accumulation of (-)-terremutin was noticed in plantlet tissues (Figure 6D). With PTA-271-pretreated plantlets, a similar trend appeared for (-)-terremutin removal from the MS medium (Figure 6C), without any apparent accumulation inside the plant tissues (Figure 6D).

## Discussion

The contribution of (*R*)-mellein and (-)-terremutin to *N. parvum* aggressiveness was strongly suspected in grapevine, considering their detection in the wood and leaves of Botryosphaeria dieback affected plants (Abou-Mansour et al., 2015) while their secreting pathogens were exclusively wood-confined (Mugnai et al., 1999). However, the role of such phytotoxins in Botryosphaeria infectious process and their potential control by beneficial microbes remain unknown. In this study, we used a *N. parvum* strain that produces both (*R*)-mellein and (-)-terremutin, as well as these purified toxins, to understand their role in the *N. parvum* aggressiveness. We also investigated the capacity of the beneficial bacterium *B. subtilis* PTA-271 to counteract Botryosphaeria dieback symptoms, and explore whether the bacterium can affect pathogen growth, detoxify pure toxins and prime grapevine immunity after pathogen infection.

Our data provide evidence that *N. parvum Np*-Bt67 which produces high amount of (-)-terremutin provoked Botryosphaeria dieback symptoms within 10 days on grapevine cuttings, including dead branch, canker and both external and internal stem necrosis (Figure 1). Interestingly, after treatment of cutting at the root level with PTA-271, the Botryosphaeria dieback symptoms were significantly reduced. The PTA-271-pretreated plants showed a reduced dead branch of 50% after *Np*-Bt67 challenge, accompanied with a strong reduction of canker and stem lesions. This study reports for the first time: (i) the expression of a severe form of Botryosphaeria dieback on Chardonnay plants in controlled conditions, and (ii) that PTA-271 seems to be a very effective bacterium to protect Chardonnay plants against a Botryosphaeria pathogen. This protective effect appears to be related to the ability of the bacterium to antagonize *N. parvum* by delaying its mycelial growth, to detoxify both (*R*)-mellein and (-)-terremutin, and to prime few defense genes including *PR2* (a β-1,3-glucanase), *NCED2* (involved in ABA synthesis) and *PAL* at systemic level after pathogen inoculation. Indeed PTA-271 was initially isolated from grapevine rhizosphere, while inducing leaf defense responses (Trotel-Aziz et al., 2008). But Santoyo et al. (2016) indicates that all of the genera described as common inhabitants of the rhizosphere, are also bacterial endophytes. Especially *Bacillus* sp. is the most commonly isolated species from all kinds of grapevine tissues including the wood of both Esca-foliar symptomatic or asymptomatic plants (Bruez et al., 2015). In this study, PTA-271 succeeds to protect grapevine. Whatever the inhabiting zone of PTA-271 or its active molecules, PTA-271 impacts were sought both on mycelium and toxins of fungal pathogen and on plant immunity.

The antagonistic activity of *B. subtilis* PTA-271 against *Np*-Bt67 (Figure 2) showed some dependency on temperature, since it is only effective at 28°C. PTA-271 could thus impact the life cycle of *N. parvum*, especially at 28°C since it clearly appears that PTA-271 grows less at 22°C while using identical bacterial densities at day 0. Thus the less fungal inhibition at 22°C might result from the fact that PTA-271 grows less at 22°C. This fungistatic effect might be explained by the release of various antifungal compounds by PTA-271, including surfactins or other lipopeptides which production was shown to depend on temperature (Ongena and Jacques, 2008; Pinto et al., 2018). Interestingly, PTA-271 can also detoxify the two main phytotoxins of *N. parvum* to different extents (Figure 3). The detoxifying activity of the bacterium seems to be more active in a nutrient rich medium for (-)-terremutin, but not for (*R*)-mellein. This suggests that (*R*)-mellein would be directly metabolized by PTA-271, while (-)-terremutin would require a co-substrate to be co-metabolized by this bacterium. This is consistent with the short latency period needed for (-)-terremutin mobilization from the medium, as already reported for some organic pesticides (Cycon and Piotrowska-Seget, 2016). It has been reported that bacteria can use root exudates such as catechin and coumarin as co-substrates to detoxify recalcitrant organic molecules *in situ* (Makova et al., 2006). It is thus speculated that grapevine and beneficial bacteria might interact together to improve detoxification process and then ensure an active protection against Botryosphaeria dieback. However, in the case of (*R*)-mellein, its detoxification rate by bacteria is characterized by a long latency phase followed by a rapid disappearance, even at a low bacterial density (10^4^ cfu/mL). This latency period would be necessary for bacteria to express its detoxifying pathways.

Our results also suggested that PTA-271 might prime the expression of some plant defense genes responsive to different phytohormone pathways (Figure 4). In leaves of control cuttings challenged with *Np*-Bt67, some genes were slightly up-regulated, especially *PR2*, *PR5*, *PR10*, as SA-responsive genes (Dufour et al., 2013; Naznin et al., 2014; Caarls et al., 2015), while the expression of *PR4*, *LOX9*, as JA/ET responsive genes (Hamiduzzaman et al., 2005; Naznin et al., 2014) remained low. This suggests that the early activation of SA-signaling during pathogen’s biotrophic phase could antagonize the expression of JA-dependent-defenses useful for grapevine once pathogen entered its necrotrophic phase as indicated by Yang et al. (2015). This could result from the pathogen strategy to overcome host defenses and thus promote disease. In the same sense, a late and weak defense’s expression has been already observed in grapevines developing Botryosphaeria dieback symptoms in vineyards (Spagnolo et al., 2014). In PTA-271-pretreated plants, *PR2* was highly primed after pathogen inoculation, and to a weakest extent for *LOX9* as JA-dependent, *GST1*, *PAL*, and *STS* associated to secondary metabolism, or *NCED2* involved in ABA biosynthesis. Interestingly, the expression of *PR2* gene is described to be regulated by various phytohormones such as SA, JA, and ET (Liu et al., 2010). Up to date, it is still unclear how the SA-induced cellular changes can influence JA-inducible responses (Caarls et al., 2015). Pretreatment with PTA-271 might thus up-regulate *PR2* expression in a JA-dependent way. *PR2* encodes a β-1,3-glucanase, which could play an important role in grapevine defense, either directly, through the degradation of pathogen cell wall, or indirectly, by releasing oligosaccharide elicitors that could induce additional plant defenses (Renault et al., 2000). Although this priming effect essentially but indisputably concerns more *PR2* following Np-Bt67 challenge, it does not counter balance the PTA-271 priming capacity toward grapevine pathogens. Indeed, primed plants usually show no enhanced expression of phenotypic defense traits, but they respond faster or more strongly following the pathogen challenge inoculation (Conrath et al., 2006; Goellner and Conrath, 2008), as observed for *PR2* with PTA-271 treated cuttings at this time point of analysis (4 dpi). Verhagen et al. (2011) also showed a PTA-271 capacity to induce slight plant leaf defense responses, but further potentiated upon *B. cinerea* challenge (from 3 to 7 dpi, using a plantlet model). We also showed (Trotel-Aziz et al., 2008) that PTA-271 can stimulate JA/ET-dependent defenses in grapevine against the necrotrophic fungus *B. cinerea*. Regarding the expression of *NCED2* primed by PTA-271 upon pathogen challenge, our data cannot exclude a possible contribution of ABA biosynthesis to an enhanced JA biosynthesis (Adie et al., 2007) that remains to be further elucidated.

Deciphering now grapevine immune response using plantlets directly exposed to (*R*)-mellein and (-)-terremutin (Figure 5), our data showed that application of (*R*)-mellein and (-)-terremutin resulted in up-regulation of the SA-responsive genes *PR1* and *GST1*, respectively (Devadas et al., 2002). *GST1* is also part of the array of defense-related genes induced in response to oxidative burst produced after pathogen infection (Bhattacharjee, 2012). Contrary to SA-responsive genes, the expression of JA/ET-responsive genes remained weak as shown previously during grapevine-*Np*-Bt67 interaction, or even down-regulated by (-)-terremutin (i.e., *PR3*). This can be supported by the fact that (-)-terremutin is a derivative of 6-methyl-SA (Guo et al., 2014) as a mobile signal easily hydrolysable to active SA (Park et al., 2007; Kumar and Klessig, 2008). It is thus tempting to correlate (-)-terremutin to the necrotrophic stage of *Np*-Bt67 lifestyle, and to speculate the mimicking of SA effect to antagonize JA-dependent defenses. In contrast, (*R*)-mellein induced both the SA-responsive *PR1* and *PR5* and to a weaker extent the JA/ET-dependent *PR4* gene. Thus, (*R*)-mellein produced by *N. parvum* might be mainly in link with the biotrophic and early necrotrophic stages of pathogen with hemibiotrophic lifestyle (Duan et al., 2014; Ross et al., 2014; Yang et al., 2015). In PTA-271 pretreated plantlets (Figure 5), both JA/ET- and SA-responsive genes were up-regulated, as well as an ABA biosynthetic gene (*NCED2*) and phenylpropanoid pathway genes (*PAL*, *STS*) in *in vitro*-plantlets. These data are in agreement with those of Trotel-Aziz et al. (2008) using the same plantlet model. However, exogenous application of (-)-terremutin and (*R*)-mellein repressed the expression of almost all of the PTA-271 up-regulated host-defense-genes. The enhanced expression of *GST1* by PTA-271 was weakly repressed by (-)-terremutin, suggesting that GST could take part to the detoxification process of (-)-terremutin or maybe in the redox regulation in SA/JA crosstalk. Some authors have indicated that overexpression of *GST1* might mediate redox changes to prevent some pathogen aggressive molecules to mimic SA-signaling to overcoming host immunity (Tada et al., 2008; Vidhyasekaran, 2015). Interestingly, up-regulation of *NCED2* by PTA-271 was not altered by fungal toxins, emphasizing the role of ABA as a central component to overcome toxin effects by a possible enhancement of JA synthesis (Adie et al., 2007; Mohr and Cahill, 2007; Spoel and Dong, 2008). Many studies reported that endogenously accumulated SA antagonizes JA-dependent defenses, thereby prioritizing SA-dependent resistance over JA-dependent defense (Pieterse et al., 2012; Van der Does et al., 2013). Indeed *PR1* was the sole gene still over-induced in PTA-271 pretreated plantlets exposed to each pure toxin. This is consistent with our hypothesis of a SA mimicking effect to antagonize the host JA-dependent defenses. Deciphering the extend of cross-communication in the hormone signaling pathways, through fine tuning of transcriptional programs, would thus enable to better understand the mechanisms contributing to grapevine basal and induced resistances to GTD pathogens. The potential roles of *GST1* overexpressed in the presence of (-)-terremutin, and of *NCED2* upregulated in the presence of PTA-271, would now merit a greater attention.

PTA-271 beneficial effect might also target grapevine detoxifying capacity on GTD-secreted phytotoxins. Control plantlets can mobilize both (*R*)-mellein and (-)-terremutin when exogenously applied at the root level (Figure 6). (*R*)-Mellein is entirely mobilized and may be accumulated *in planta* in its native chemical form, while (-)-terremutin was partly mobilized and was not accumulated *in planta*. In contrast, in PTA-271-pretreated plantlets, only (*R*)-mellein mobilization was slightly reduced. Treatment with PTA-271 might thus slow down the (*R*)-mellein uptake by grapevine plantlets. The distinct chemical structures of each toxin still remain to be investigated (i.e., toxin conjugates), as well as the mechanisms slowing down (*R*)-mellein entry in plantlets, to better understand how PTA-271 might exert its beneficial effects on grapevine’s detoxifying capacity.

## Conclusion

Altogether, our results provide evidences that (-)-terremutin and (*R*)-mellein are usefull molecules for *N. parvum* that can secrete them inside the host to fully express its virulent character. Once inside the plant (-)-terremutin and (*R*)-mellein may reprogram grapevine immunity enabling the pathogen to overcome host defenses and thus promote disease. However, the beneficial bacterium PTA-271 significantly attenuated the Botryosphaeria dieback symptoms, by antagonizing *N. parvum* growth, inducing plant systemic resistance as shown by the strong *PR2* priming among the few host defense responses in the tested time point, and detoxifying both (*R*)-mellein and (-)-terremutin produced by *Np*-Bt67.

## Author Contributions

PTA planned and designed the research, performed most of the experiments, analyzed the data, and wrote the manuscript with the contributions and discussion from AA, FF, EAM, and CC. FF, AA, EAM, and CC validated the planned research and gave their expertise for all steps of this work. EAM prepared all purified toxins for the experiments. BC performed most of the qRT-PCR experiments and prepared grapevine plantlets. FR ensured the quality of qRT-PCR analysis and data.

## Conflict of Interest Statement

The authors declare that the research was conducted in the absence of any commercial or financial relationships that could be construed as a potential conflict of interest.

## Supplementary Material

Figure S1 Toxicity assessment of (*R*)-mellein and (-)-terremutin toward *B. subtilis* PTA-271 (Bs) in 24 h. PTA-271 was sprayed on PDA plates containing one central phytotoxin drop (5 μL) from 0 to 60 mg/L for (*R*)-mellein (M0 to M60) or (-)-terremutin (T0 to T60).

Figure S2 Toxicity assessment of (*R*)-mellein and (-)-terremutin toward*in vitro-*plantlets. Eight weeks old plantlets were exposed to MS medium containing (*R*)-mellein or (-)-terremutin at concentrations ranging from 0 to 1500 μg/L. Three days post-exposure (dpe) to phytotoxin, toxicity was assessed through maximum quantum yield of photosynthesis (Fv/Fm), fresh weight and pigment concentrations. The maximum photosynthetic capacity of the plants was obtained by measuring the Fv/Fm parameter given by a PAM-Pulse Amplitude Modulated fluorimeter equipped with the Modfluor v2.00 software (Hansath, London, United Kingdom) according to the recommendations of Genty et al. (1990). The content of chlorophylls a, b, and carotenoids was obtained by colorimetric assay (spectrophotometry at 470, 652.4, and 665.2 nm) after pigments extraction in pure methanol (20 min at 65°C) and quantified according to Wellburn (1994) formulas. Data are means ± SD of three independent experiments, each with four replicates. None bars were headed with asterisks, indicating none significant differences (Multiple Comparison procedures with Tukey’s test, *P* < 0.05).

Figure S3 *B. subtilis* PTA-271 primes some defense-related genes in leaves of grapevine cuttings after infection with the *N. parvum* strain *Np*-Bt67. Legend as in Figure 4. Three-color scale as in Figure 5, with deep red corresponding to an induction factor of 23.71 or more, and dark blue corresponding to a 0.55-fold induction or less.

Figure S4 (*R*)-Mellein and (-)-terremutin repress the *B. subtilis*-PTA-271-induced immune responses in grapevine plantlets. Different letters indicate significant differences. Legend as in Figure 5.

Table S1 Primer sequences used for qRT-PCR analysis of defense-related genes.

## References

[B1] Abou-Mansour E. Débieux J. L. Ramírez-Suero M. Bénard-Gellon M. Magnin-Robert M. Spagnolo A. (2015). Phytotoxic metabolites from *Neofusicoccum parvum*, a pathogen of Botryosphaeria dieback of grapevine. *Phytochemistry* 115 207–215. 10.1016/j.phytochem.2015.01.012 25747381

[B2] Adie B. A. T. Pérez-Pérez J. Pérez-Pérez M. M. Godoy M. Sanchez-Serrano J.-J. Schmelz E. A. (2007). ABA is an essential signal for plant resistance to pathogens affecting JA biosynthesis and the activation of defenses in *Arabidopsis*. *Plant Cell* 19 1665–1681. 10.1105/tpc.106.048041 17513501 PMC1913739

[B3] Andolfi A. Mugnai L. Luque J. Surico G. Cimmino A. Evidente A. (2011). Phytotoxins produced by fungi associated with grapevine trunk diseases. *Toxins* 3 1569–1605. 10.3390/toxins3121569 22295177 PMC3268457

[B4] Aziz A. Verhagen B. Magnin-Robert M. Couderchet M. Clément C. Jeandet P. (2016). Effectiveness of beneficial bacteria to promote systemic resistance of grapevine towards gray mold as related to phytoalexin production in vineyards. *Plant Soil* 405 141–153. 10.1007/s11104-015-2783-z

[B5] Bakker P. A. H. M. Doornbos R. F. Zamioudis C. Berendsen R. L. Pieterse C. M. J. (2013). Induced systemic resistance and the rhizosphere microbiome. *Plant Pathol. J.* 29 136–143. 10.5423/PPJ.SI.07.2012.0111 25288940 PMC4174772

[B6] Benhamou N. le Floch G. Vallance J. Gerbore J. Grizard D. Rey P. (2012). *Pythium oligandrum*: an example of opportunistic success. *Microbiology* 158 2679–2694. 10.1099/mic.0.061457-0 22977087

[B7] Bertsch C. Ramirez-Suero M. Magnin-Robert M. Larignon P. Chong J. Abou-Mansour E. (2013). Grapevine trunk diseases: complex and still poorly understood. *Plant Pathol.* 62 243–265. 10.1111/j.1365-3059.2012.02674.x

[B8] Bhattacharjee S. (2012). The language of reactive oxygen species signaling in plants. *J. Bot.* 2012:985298. 10.1155/2012/985298 19704468

[B9] Bruez E. Haidar R. Alou M. T. Vallance J. Bertsch C. Mazet F. (2015). Bacteria in a wood fungal disease: characterization of bacterial communities in wood tissues of esca-foliar symptomatic and asymptomatic grapevines. *Front. Microb.* 6:1137. 10.3389/fmicb.2015.01137 26579076 PMC4621878

[B10] Caarls L. Pieterse C. M. J. Van Wees S. C. M. (2015). How salicylic acid takes transcriptional control over jasmonic acid signaling. *Front. Plant Sci.* 6:170. 10.3389/fpls.2015.00170 25859250 PMC4373269

[B11] Cabras A. Mannoni M. A. Serra S. Andolfi A. Fiore M. (2006). Occurrence, isolation and biological activity of phytotoxic metabolites produced *in vitro* by *Sphaeropsis sapinea*, pathogenic fungus of *Pinus radiata*. *Eur. J. Plant Pathol.* 115 187–193. 10.1007/s10658-006-9006-7

[B12] Chen A. J. Varga J. Frisvad J. C. Jiang X. Z. Samson R. A. (2016). Polyphasic taxonomy of *Aspergillus* section Cervini. *Stud. Mycol.* 85 65–89. 10.1016/j.simyco.2016.11.001 28050054 PMC5192051

[B13] Chooi Y. H. Krill C. Barrow R. A. Chen S. Trengove R. Oliver R. P. (2015). An in planta-expressed polyketide synthase produces (R)-mellein in the wheat pathogen *Parastagonospora nodorum*. *Appl. Environ. Microbiol.* 81 177–186. 10.1128/AEM.02745-14 25326302 PMC4272741

[B14] Christen D. Tharin M. Perrin-Cherioux S. Abou-Mansour E. Tabacchi R. Défago G. (2005). Transformation of Eutypa dieback and Esca disease pathogen toxins by antagonistic fungal strains reveals a second Detoxification pathway not present in *Vitis vinifera*. *J. Agric. Food Chem.* 53 7043–7051. 10.1021/jf050863h 16131109

[B15] Conrath U. Beckers G. J. M. Flors V. Garcia-Agustin P. Jakab G. Mauch F. (2006). Priming: getting ready for battle. *Mol. Plant Microbe Interact.* 19 1062–1071. 10.1094/MPMI-19-1062 17022170

[B16] Cycon M. Piotrowska-Seget Z. (2016). Pyrethroid-degrading microorganisms and their potential for the bioremediation of contaminated soils : a review. *Front. Plant Sci.* 7:1463. 10.3389/fmicb.2016.01463 27695449 PMC5023672

[B17] Dalmais B. Schumacher J. Moraga J. Le Pêcheur P. Tudzynski B. Gonzalez Collado I. (2011). The *Botrytis cinerea* phytotoxin botcinic acid requires two polyketide synthases for production and has a redundant role in virulence with botrydial. *Mol. Plant Pathol.* 12 564–579. 10.1111/j.1364-3703.2010.00692.x 21722295 PMC6640383

[B18] Devadas S. K. Enyedi A. Raina R. (2002). The Arabidopsis *hrl1* mutation reveals novel overlapping roles for salicylic acid, jasmonic acid and ethylene signalling in cell death and defence against pathogens. *Plant J.* 30 467–480. 10.1046/j.1365-313X.2002.01300.x 12028576

[B19] Di Marco S. Osti F. Cesari A. (2004). Experiments on the control of esca by *Trichoderma*. *Phytopathol. Mediterr.* 43 108–115. 10.1007/s00726-011-1126-5 22038182

[B20] Di Marco S. Osti F. Roberti R. Calzarano F. Cesari A. (2002). Attività di specie di *Trichoderma* nei confronti di *Phaeomoniella chlamydospora*, patogeno associato al mal dell’esca della vite. *Atti Giornate Fitopatol.* 731 419–424.

[B21] Ding W. Lei C. He Q. Zhang Q. Bi Y. Liu W. (2010). Insights into bacterial 6-methylsalicylic acid synthase and its engineering to orsellinic acid synthase for spirotetronate generation. *Chem. Biol.* 17 495–503. 10.1016/j.chembiol.2010.04.009 20534347

[B22] Djoukeng J. D. Polli S. Larignon P. Abou-Mansour E. (2009). Identification of phytotoxins from *Botryosphaeria obtusa*, a pathogen of black dead arm disease of grapevine. *Eur. J. Plant Pathol.* 124 303–308. 10.1007/s10658-008-9419-6

[B23] Duan L. Liu H. Li X. Xiao J. Wang S. (2014). Multiple phytohormones and phytoalexins are involved in disease resistance to *Magnaporthe oryzae* invaded from roots in rice. *Physiol. Plant.* 152 486–500. 10.1111/ppl.12192 24684436

[B24] Dufour M. C. Lambert C. Bouscaut J. Mérillon J. M. Corio-Costet M. F. (2013). Benzothiadiazole-primed defence responses and enhanced differential expression of defence genes in *Vitis vinifera* infected with biotrophic pathogens *Erysiphe necator* and *Plasmopara viticola*. *Plant Pathol.* 62 370–382. 10.1111/j.1365-3059.2012.02628.x

[B25] Espinosa J. G. Briceño E. X. Chávez E. R. Úrbez-Torres J. R. Latorre B. A. (2009). Neofusicoccum spp. associated with stem canker and dieback of blueberry in Chile. *Plant Dis.* 93 1187–1194. 10.1094/PDIS-93-11-1187 30754575

[B26] Evidente A. Punzo B. Andolfi A. Cimmino A. Melck D. Luque J. (2010). Lipophilic phytotoxins produced by *Neofusicoccum parvum*, a grapevine canker agent. *Phytopathol. Mediterr.* 49 74–79.

[B27] Fontaine F. Pinto C. Vallet J. Clément C. Gomes A. C. Spagnolo A. (2015). The effects of grapevine trunk diseases (GTDs) on vine physiology. *Eur. J. Plant Pathol.* 144 707–721. 10.1007/s10658-015-0770-0

[B28] Genty B. Harbinson J. Briantais J. M. Baker N. R. (1990). The relationship between non-photochemical quenching of fluorescence and the rate of photosystem II photochemistry in leaves. *Photosynth. Res.* 25 249–257. 10.1007/BF00033166 24420355

[B29] Goellner K. Conrath U. (2008). Priming: it’s all the world to induced disease resistance. *Eur. J. Plant Pathol.* 121 233–242. 10.1007/s10658-007-9251-4

[B30] Gressler M. Meyer F. Heine D. Hortschansky P. Hertweck C. Brock M. (2015). Phytotoxin production in *Aspergillus terreus* is regulated by independent environmental signals. *eLife Sci.* 4:e07861. 10.7554/eLife.07861 26173180 PMC4528345

[B31] Gruau C. Trotel-Aziz P. Villaume S. Rabenoelina F. Clément C. Baillieul F. (2015). *Pseudomonas fluorescens* PTA-CT2 triggers local and systemic immune response against *Botrytis cinerea* in grapevine. *Mol. Plant Microbe Interact.* 28 1117–1129. 10.1094/MPMI-04-15-0092-R 26075828

[B32] Guo C.-J. Sun W.-W. Bruno K. S. Wang C. C. C. (2014). Molecular genetic characterization of terreic acid pathway in *Aspergillus terreus*. *Org. Lett.* 16 5250–5253. 10.1021/ol502242a 25265334 PMC4201328

[B33] Haidar R. Deschamps A. Roudet J. Calvo-Garrido C. Bruez E. Rey P. (2016). Multi-organ screening of efficient bacterial control agents against two major pathogens of grapevine. *Biol. Control* 92 55–65. 10.1016/j.biocontrol.2015.09.003

[B34] Halleen F. Fourie P. H. Lombard P. J. (2010). Protection of grapevine pruning wounds against *Eutypa lata* by biological and chemical methods. *S. Afr. J. Enol. Vitic.* 31 125–132.

[B35] Hamiduzzaman Md Jakab G. Barnavon L. Neuhaus J.-M. Mauch-Mani B. (2005). β-aminobutyric acid-induced resistance against downy mildew in grapevine acts through the potentiation of callose formation and jasmonic acid signaling. *Mol. Plant Microbe Interact.* 18 819–829. 10.1094/MPMI-18-0819 16134894

[B36] Han H. Yang Y. Olesen S. H. Becker A. Betzi S. Schönbrunn E. (2010). The fungal product terreic acid is a covalent inhibitor of the bacterial cell wall biosynthetic enzyme UDP-N-acetylglucosamine 1-carboxyvinyltransferase (MurA). *Biochemistry* 49 4276–4282. 10.1021/bi100365b 20392080 PMC2884014

[B37] John S. Wicks T. J. Hunt J. S. Lorimer M. F. Oakey H. Scott E. S. (2005). Protection of grapevine pruning wounds from infection by *Eutypa lata* using *Trichoderma harzianum* and *Fusarium lateritium*. *Australas. Plant Pathol.* 34 569–575. 10.1071/AP05075

[B38] John S. Wicks T. J. Hunt J. S. Scott E. S. (2008). Colonisation of grapevine wood by *Trichoderma harzianum* and *Eutypa lata*. *Aust. J. Grape Wine Res.* 14 18–24. 10.1111/j.1755-0238.2008.00003.x

[B39] Kawakami Y. Hartman S. E. Kinoshita E. Suzuki H. Kitaura J. Yao L. (1999). Terreic acid, a quinone epoxide inhibitor of Bruton’s tyrosine kinase. *Proc. Natl. Acad. Sci. U.S.A.* 96 2227–2232. 10.1073/pnas.96.5.2227 10051623 PMC26765

[B40] Keller B. Winzeler H. Winzeler M. Fried P. M. (1994). Differential sensitivity of wheat embryos against extracts containing toxins of *Septoria nodorum*-first steps towards *in-vitro* selection. *J. Phytopathol.* 141 233–240. 10.1111/j.1439-0434.1994.tb01466.x

[B41] Kotze C. Van Niekerk J. Mostert L. Halleen F. Fourie P. (2011). Evaluation of biocontrol agents for grapevine pruning wound protection against trunk pathogen infection. *Phytopathol. Mediterr.* 50 S247–S263.

[B42] Kumar D. Klessig D. F. (2008). The search for the salicylic acid receptor led to discovery of the SAR signal receptor. *Plant Signal. Behav.* 3 691–692. 10.4161/psb.3.9.5844 19704829 PMC2634560

[B43] Larignon P. Spagnolo A. Bertsch C. Fontaine F. (2015). First report of young grapevine decline caused by *Neofusicoccum parvum* in France. *Plant Dis.* 99:1859 10.1094/PDIS-03-15-0280-PDN

[B44] Laveau C. Letouze A. Louvet G. Bastien S. Guérin-Dubrana L. (2009). Differential aggressiveness of fungi implicated in esca and associated diseases of grapevine in France. *Phytopathol. Mediterr.* 48 32–46.

[B45] Lebon G. Duchêne E. Brun O. Clément C. (2005). Phenology of flowering and starch accumulation in grape (*Vitis vinifera* L.) cuttings and vines. *Ann. Bot.* 95 943–948. 10.1093/aob/mci108 15749750 PMC4246755

[B46] Liu B. Xue X. Cui S. Zhang X. Han Q. Zhu L. (2010). Cloning and characterization of a wheat β-1,3-glucanase gene induced by the stripe rust pathogen *Puccinia striiformis* f. sp. *Tritici. Mol. Biol. Rep.* 37 1045–1052. 10.1007/s11033-009-9823-9 19757158

[B47] Magnin-Robert M. Letousey P. Spagnolo A. Rabenoelina F. Jacquens L. Mercier L. (2011). Leaf strip of esca induces alteration of photosynthesis and defence reactions in presymptomatic leaves. *Funct. Plant Biol.* 38 856–866. 10.1071/FP11083 32480943

[B48] Magnin-Robert M. Spagnolo A. Boulanger A. Joyeux C. Clément C. Abou-Mansour E. (2016). Changes in plant metabolism and accumulation of fungal metabolites in response to Esca proper and apoplexy expression in the whole grapevine. *Phytopathology* 16 541–553. 10.1094/PHYTO-09-15-0207-R 26882851

[B49] Magnin-Robert M. Trotel-Aziz P. Quantinet D. Biagianti S. Aziz A. (2007). Biological control of *Botrytis cinerea* by selected grapevine-associated bacteria and stimulation of chitinase and β-1,3 glucanase activities under field conditions. *Eur. J. Plant Pathol.* 118 43–57. 10.1007/s10658-007-9111-2

[B50] Makova M. Dowling D. Macek T. (2006). *Phytoremediation and Rhizoremediation*, eds Hofman M. Anné J. (Dordrecht: Springer), 1–299. 10.1007/978-1-4020-4999-4

[B51] McMahan G. Yeh W. Marshall M. N. Olsen M. Sananikone S. Wu J. Y. (2001). Characterizing the production of a wild-type and benomyl-resistant *Fusarium lateritium* for biocontrol of *Eutypa lata* on grapevine. *J. Ind. Microbiol. Biotechnol.* 26 151–155. 10.1038/sj.jim.7000099 11420655

[B52] Möbius N. Hertweck C. (2009). Fungal phytotoxins as mediators of virulence. *Curr. Opin. Plant Biol.* 12 390–398. 10.1016/j.pbi.2009.06.004 19608453

[B53] Mohr P. G. Cahill D. M. (2007). Suppression by ABA of salicylic acid and lignin accumulation and the expression of multiple genes, in *Arabidopsis* infected with *Pseudomonas syringae* pv. *tomato*. *Funct. Integr. Genomics* 7 181–191. 10.1007/s10142-006-0041-4 17149585

[B54] Mondello V. Songy A. Battiston E. Pinto C. Coppin C. Trotel-Aziz P. (2018). Grapevine trunk diseases: a review of fifteen years of trials for their control with chemicals and biocontrol agents. *Plant Dis.* 102 1189–1217. 10.1094/PDIS-08-17-1181-FE 30673583

[B55] Mugnai L. Graniti A. Surico G. (1999). Esca (black measles) and brown wood-streaking: two old and elusive diseases of grapevines. *Plant Dis.* 83 404–418. 10.1094/PDIS.1999.83.5.404 30845530

[B56] Naznin H. A. Kiyohara D. Kimura M. Miyazawa M. Shimizu M. Hyakumachi M. (2014). Systemic resistance induced by volatile organic compounds emitted by plant growth-promoting fungi in *Arabidopsis thaliana*. *PLoS One* 9:e86882. 10.1371/journal.pone.0086882 24475190 PMC3903595

[B57] Ongena M. Jacques P. (2008). *Bacillus* lipopeptides: versatile weapons for plant disease biocontrol. *Trends Microbiol.* 16 115–125. 10.1016/j.tim.2007.12.009 18289856

[B58] Parisi A. Piattelli M. Tringali C. Di San Lio G. M. (1993). Identification of the phytotoxin mellein in culture fluids of *Phoma tracheiphila*. *Phytochemistry* 32 865–867. 10.1016/0031-9422(93)85221-C

[B59] Park S. W. Kaimoyo E. Kumar D. Mosher S. Klessig D. F. (2007). Methyl salicylate is a critical mobile signal for plant systemic acquired resistance. *Science* 318 113–116. 10.1126/science.1147113 17916738

[B60] Pieterse C. M. J. Van der Does D. Zamioudis C. Leon-Reyes A. Van Wees S. C. M. (2012). Hormonal modulation of plant immunity. *Annu. Rev. Cell Dev. Biol.* 28 489–521. 10.1146/annurev-cellbio-092910-154055 22559264

[B61] Pinto C. Pinho D. Sousa S. Pinheiro M. Egas C. Gomes A. C. (2014). Unravelling the diversity of grapevine microbiome. *PLoS One* 9:e85622. 10.1371/journal.pone.0085622 24454903 PMC3894198

[B62] Pinto C. Sousa S. Froufe H. Egas C. Clément C. Fontaine F. (2018). Draft genome sequence of *Bacillus amyloliquefaciens* subsp. *plantarum strain Fito_F*321, an endophyte microorganism from *Vitis vinifera* with biocontrol potential. *Stand. Genomic Sci.* 13:30. 10.1186/s40793-018-0327-x 30410642 PMC6211603

[B63] Pusztahelyi T. Holb I. J. Pocsi I. (2015). Secondary metabolites in fungus-plant interactions. *Front. Plant Sci.* 6:573. 10.3389/fpls.2015.00573 26300892 PMC4527079

[B64] Ramírez-Suero M. Bénard-Gellon M. Chong J. Laloue H. Stempien E. Abou-Mansour E. (2014). Extracellular compounds produced by fungi associated with Botryosphaeria dieback induce differential defence gene expression patterns and necrosis in *Vitis vinifera* cv chardonnay cells. *Protoplasma* 251 1417–1426. 10.1007/s00709-014-0643-y 24752796

[B65] Reis P. Magnin-Robert M. Nascimento T. Spagnolo A. Abou-Mansour E. Fioretti C. (2016). Reproducing botryospaeria dieback foliar symptoms in a simple model system. *Plant Dis.* 100 1071–1079. 10.1094/PDIS-10-15-1194-RE 30682279

[B66] Renault A. S. Deloire A. Letinois I. Kraeva E. Tesniere C. Ageorges A. (2000). β-1,3-glucanase gene expression in grapevine leaves as a response to infection with *Botrytis cinerea*. *Am. J. Enol. Vitic.* 51 81–87. 10.1093/jxb/ert351 24170740

[B67] Rezgui A. Ben Ghnaya-Chakroun A. Vallance J. Bruez E. Hajlaoui M. R. Sadfi-Zouaoui N. (2016). Endophytic bacteria with antagonistic traits inhabit the wood tissues of grapevines from Tunisian vineyards. *Biol. Control* 99 28–37. 10.1016/j.biocontrol.2016.04.005

[B68] Ross C. Opel V. Scherlach K. Hertweck C. (2014). Biosynthesis of antifungal and antibacterial polyketides by *Burkholderia gladioli* in coculture with *Rhizopus microsporus*. *Mycoses* 57 48–55. 10.1111/myc.12246 25250879

[B69] Santoyo G. Moreno-Hagelsieb G. Orozco-Mosqueda C. Glick B. R. (2016). Plant growth-promoting bacterial endophytes. *Microbiol. Res.* 183 92–99. 10.1016/j.micres.2015.11.008 26805622

[B70] Schmidt C. S. Lorenz D. Wolf G. A. (2001). Biological control of the grapevine dieback fungus *Eutypa lata* I: screening of bacterial antagonists. *J. Phytopathol.* 149 427–435. 10.1046/j.1439-0434.2001.00658.x

[B71] Schroeckh V. Scherlach K. Nützmann H.-W. Shelest E. Schmidt-Heck W. Schuemann J. (2009). Intimate bacterial-fungal interaction triggers biosynthesis of archetypal polyketides in *Aspergillus nidulans*. *Proc. Natl. Acad. Sci. U.S.A.* 106 14558–14563. 10.1073/pnas.0901870106 19666480 PMC2732885

[B72] Spagnolo A. Magnin-Robert M. Alayi T. D. Cilindre C. Shaeffer-Reiss C. Van Dorsselaer A. (2014). Differential responses of three grapevine cultivars to Botryosphaeria dieback. *Phytopathology* 104 1021–1035. 10.1094/PHYTO-01-14-0007-R 24724741

[B73] Spagnolo A. Magnin-Robert M. Dilezitoko Alayi T. Cilindre C. Mercier L. Schaeler-Reiss C. (2012). Physiological changes in green stems of *Vitis vinifera* L. cv. chardonnay in response to esca proper and apoplexy revealed by proteomic and transcriptomic analyses. *J. Proteome Res.* 11 461–475. 10.1021/pr200892g 22050466

[B74] Spagnolo A. Mondello V. Larignon P. Villaume S. Rabenoelina F. Clément C. (2017). Defense responses in grapevine (cv. Mourvèdre) after inoculation with the Botryosphaeria dieback pathogens *Neofusicoccum parvum* and *Diplodia seriata* and their relationship with flowering. *Int. J. Mol. Sci.* 18 393–405. 10.3390/ijms18020393 28208805 PMC5343928

[B75] Spoel S. H. Dong X. (2008). How do plants achieve immunity? Defence without specialized immune cells. *Nat. Rev. Immunol.* 12 89–100. 10.1038/nri3141 22273771

[B76] Surico G. Mugnai L. Marchi G. (2006). Older and more recent observations on esca: a critical review. *Phytopathol. Mediterr.* 45 68–86.

[B77] Tada Y. Spoel S. H. Pajerowska-Mukhtar K. Mou Z. Song J. Wang C. (2008). Plant immunity requires conformational charges of NPR1 via S-nitrosylation and thioredoxins. *Science* 321 5891–5900. 10.1126/science.1156970 18635760 PMC3833675

[B78] Trotel-Aziz P. Couderchet M. Biagianti S. Aziz A. (2008). Characterization of new bacterial biocontrol agents *Acinetobac*ter, *Bacillus, Pantoea* and *Pseudomonas* spp. mediating grapevine resistance against *Botrytis cinerea*. *Environ. Exper. Bot.* 64 21–32. 10.1016/j.envexpbot.2007.12.009

[B79] Uppalapati S. R. Ishiga Y. Wangdi T. Kunkel B. N. Anand A. Mysore K. S. (2007). The phytotoxin coronatine contributes to pathogen fitness and is required for suppression of salicylic acid accumulation in tomato inoculated with *Pseudomonas syringae* pv. tomato DC3000. *Mol. Plant Microbe Interact.* 20 955–965. 10.1094/MPMI-20-8-0955 17722699

[B80] Úrbez-Torres J. R. (2011). The status of botryosphaeriaceae species infecting grapevines. *Phytopathol. Mediterr.* 50 5–45.

[B81] Van der Does D. Leon-Reyes A. Koornneef A. Van Verk M. C. Rodenburg N. Pauwels L. (2013). Salicylic acid suppresses jasmonic acid signaling downstream of SCFCOI1-JAZ by targeting GCC promoter motifs via transcription factor ORA59. *Plant Cell* 25 744–761. 10.1105/tpc.112.108548 23435661 PMC3608790

[B82] Venkatasubbaiah P. Sutton T. B. Chilton W. S. (1991). Effect of phytotoxins produced by *Botryosphaeria obtusa*, the cause of black rot of apple fruit and frogeye leaf spot. *Phytopathology* 81 243–247. 10.1094/Phyto-81-243

[B83] Venkatasubbaiah P. Van Dyke C. G. Chilton W. S. (1992). Phytotoxic metabolites of *Phoma sorghina*, a new foliar pathogen of pokeweed. *Mycologia* 84 715–723. 10.1080/00275514.1992.12026197

[B84] Verhagen B. Trotel-Aziz P. Jeandet P. Baillieul F. Aziz A. (2011). Improved resistance against *Botrytis cinerea* by grapevine-associated bacteria that induce a prime oxidative burst and phytoalexin production. *Phytopathology* 101 768–777. 10.1094/PHYTO-09-10-0242 21425931

[B85] Verhagen B. W. M. Glazebrook J. Zhu T. Chang H. S. van Loon L. C. Pieterse C. M. J. (2004). The transcriptome of rhizobacteria-induced systemic resistance in *Arabidopsis*. *Mol. Plant Microbe Interact.* 17 895–908. 10.1094/MPMI.2004.17.8.895 15305611

[B86] Vidhyasekaran P. (2015). “Jasmonate signaling system in plant innate immunity,” in *Plant Hormone Signaling Systems in Plant Innate Immunity*, eds Hakeem K. R. Akhtar M. S. (New York, NY: Springer), 123–194.

[B87] Wellburn A. R. (1994). The spectral determination of chlorophylls a and b, as well as total carotenoids, using various solvents with spectrophotometers of different resolution. *J. Plant Physiol.* 144 307–313. 10.1016/S0176-1617(11)81192-2

[B88] Yacoub A. Gerbore J. Magnin N. Chambon P. Dufour M.-C. Corio-Costet M. F. (2016). Ability of *Pythium oligandrum* strains to protect *Vitis vinifera* L., by inducing plant resistance against *Phaeomoniella chlamydospora*, a pathogen involved in Esca, a grapevine trunk disease. *Biol. Control* 92 7–16. 10.1016/j.biocontrol.2015.08.005

[B89] Yamamoto H. Moriyama K. Jinnouchi H. Yagishita K. (1980). Studies on terreic acid. *Jpn. J. Antibiot.* 33 320–328. 7190624

[B90] Yang Y.-X. Ahammed G. J. Wu C. Fan S. Y. Zhou Y.-H. (2015). Crosstalk among jasmonate, salicylate and ethylene signaling pathways in plant disease and immune responses. *Curr. Protein Pept. Sci.* 16 450–461. 10.2174/1389203716666150330141638 25824390

[B91] Zaehle C. Gressler M. Shelest E. Geib E. Hertweck C. Brock M. (2014). Terrein biosynthesis in *Aspergillus turrets* and its impact on phytotoxicity. *Chem. Biol.* 21 719–731. 10.1016/j.chembiol.2014.03.010 24816227

[B92] Zarraonaindia I. Owens S. M. Weisenhorn P. West K. Hampton-Marcell J. Lax S. (2015). The soil microbiome influences grapevine-associated microbiota. *mBio* 6:e02527. 10.1128/mBio.02527-14 25805735 PMC4453523

[B93] Zeilinger S. Garcia-Estrada C. Martin J.-F. (2015). “Fungal secondary metabolites in the OMICS era,” in *Biosynthesis and Molecular Genetics of Fungal Secondary Metabolites* Vol. 2 eds Zeilinger S. Martin J.-F. Garcia-Estrada C. (New York, NY: Springer), 1–12. 10.1007/978-1-4939-2531-5

[B94] Zeilinger S. Gruber S. Bansal R. Mukherje P. K. (2016). Secondary metabolism in *Trichoderma* - chemistry meets genomics. *Fungal Biol. Rev.* 30 74–90. 10.1016/j.fbr.2016.05.001 15381847

