# Supplementary figures and images for: Bacillus subtilis PTA-271 Counteracts Botryosphaeria Dieback in Grapevine, Triggering Immune Responses and Detoxification of Fungal Phytotoxins
Source: Front Plant Sci. 2019 Jan 24;10:25. doi: 10.3389/fpls.2019.00025 (PMC6354549; doi:10.3389/fpls.2019.00025)

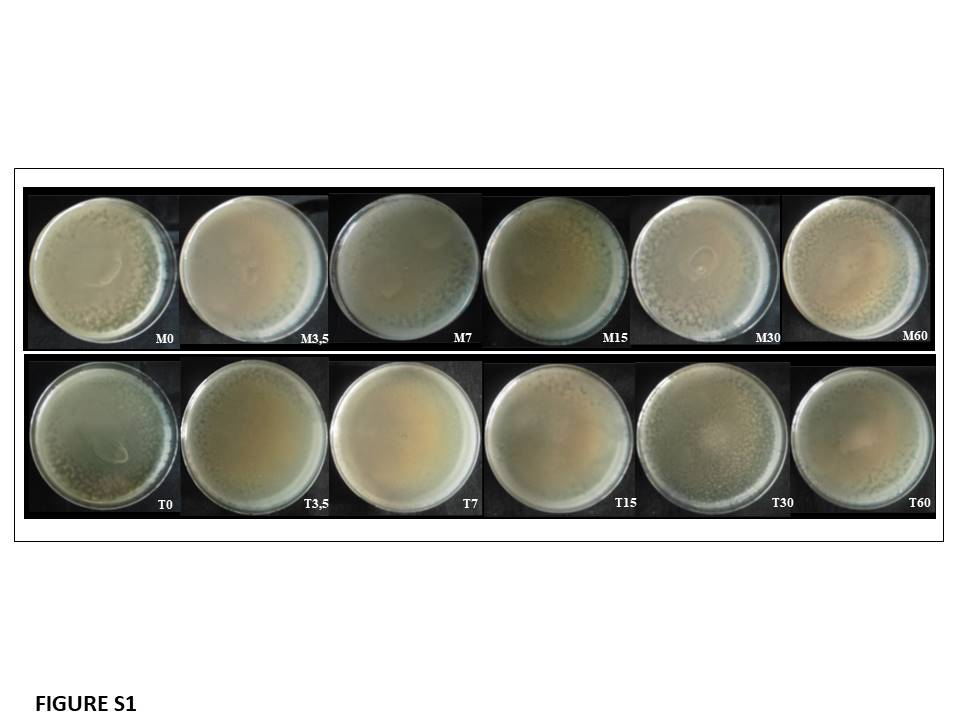

Supplement: Figure S1 — Toxicity assessment of (R)-mellein and (-)-terremutin toward B. subtilis PTA-271 (Bs) in 24 h. PTA-271 was sprayed on PDA plates containing one central phytotoxin drop (5 μL) from 0 to 60 mg/L for (R)-mellein (M0 to M60) or (-)-terremutin (T0 to T60). [file Image_1.JPEG]

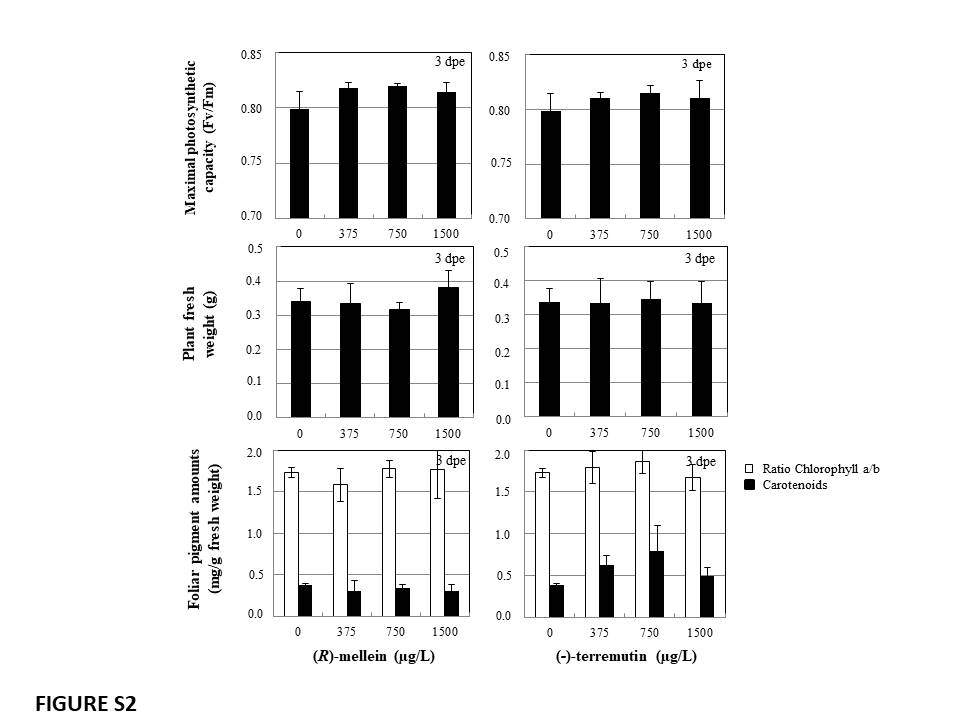

Supplement: Figure S2 — Toxicity assessment of (R)-mellein and (-)-terremutin towardin vitro-plantlets. Eight weeks old plantlets were exposed to MS medium containing (R)-mellein or (-)-terremutin at concentrations ranging from 0 to 1500 μg/L. Three days post-exposure (dpe) to phytotoxin, toxicity was assessed through maximum quantum yield of photosynthesis (Fv/Fm), fresh weight and pigment concentrations. The maximum photosynthetic capacity of the plants was obtained by measuring the Fv/Fm parameter given by a PAM-Pulse Amplitude Modulated fluorimeter equipped with the Modfluor v2.00 software (Hansath, London, United Kingdom) according to the recommendations of Genty et al. (1990). The content of chlorophylls a, b, and carotenoids was obtained by colorimetric assay (spectrophotometry at 470, 652.4, and 665.2 nm) after pigments extraction in pure methanol (20 min at 65°C) and quantified according to Wellburn (1994) formulas. Data are means ± SD of three independent experiments, each with four replicates. None bars were headed with asterisks, indicating none significant differences (Multiple Comparison procedures with Tukey’s test, P < 0.05). [file Image_2.JPEG]

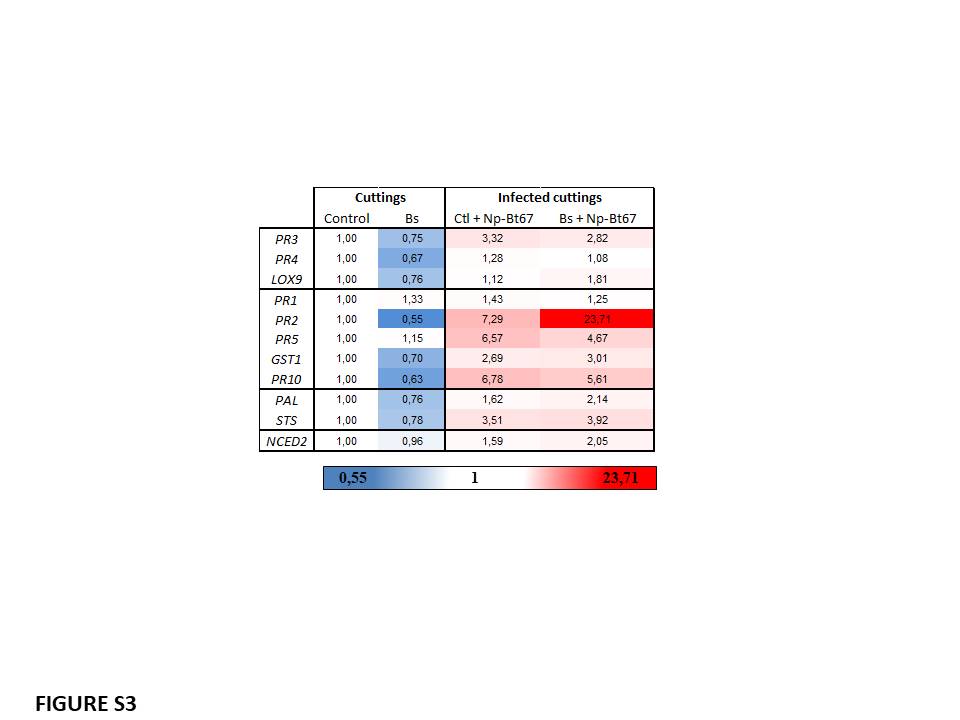

Supplement: Figure S3 — B. subtilis PTA-271 primes some defense-related genes in leaves of grapevine cuttings after infection with the N. parvum strain Np-Bt67. Legend as in Figure 4. Three-color scale as in Figure 5, with deep red corresponding to an induction factor of 23.71 or more, and dark blue corresponding to a 0.55-fold induction or less. [file Image_3.JPEG]

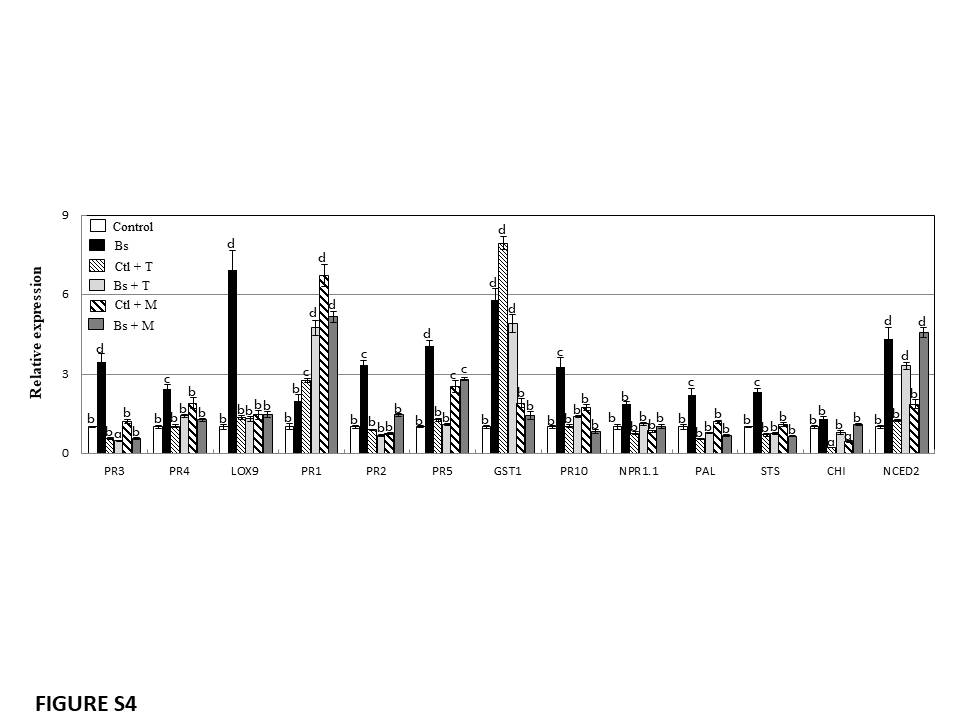

Supplement: Figure S4 — (R)-Mellein and (-)-terremutin repress the B. subtilis-PTA-271-induced immune responses in grapevine plantlets. Different letters indicate significant differences. Legend as in Figure 5. [file Image_4.JPEG]
