# Supplementary material for: Bacillus subtilis PTA-271 Counteracts Botryosphaeria Dieback in Grapevine, Triggering Immune Responses and Detoxification of Fungal Phytotoxins
Source: Front Plant Sci. 2019 Jan 24;10:25. doi: 10.3389/fpls.2019.00025 (PMC6354549; doi:10.3389/fpls.2019.00025)
Supplement: Table S1 — Primer sequences used for qRT-PCR analysis of defense-related genes. [file Table_1.docx]

**TABLE S1**: Primer sequences used for qRT-PCR analysis of defense-related genes.

| **Gene** | **Name** | **Accession number^1^** | **Forward Primer (5’-3’)** | | **Reverse primer (5’-3’)** | **Annealing temperature (°C)** | **Amplicon size (bp)** | **Efficiency of primers pairs (%)** |
| --- | --- | --- | --- | --- | --- | --- | --- | --- |
| *60RSP* | 60S ribosomal protein L18 | XM_002270599**^1^** | | ATCTACCTCAAGCTCCTAGTC | CAATCTTGTCCTCCTTTCCT | 60 | 166 | 100.0 |
| *EF1* | elongation factor 1-alpha | XM_002284888**^1^** | | AACCAAAATATCCGGAGTAAAAGA | GAACTGGGTGCTTGATAGGC | 60 | 164 | 100.0 |
| *PR3* | class IV chitinase (CHI4C) | \| NM_001281244**^1^** \| \| --- \| | | TCGAATGCGATGGTGGAAA | TCCCCTGTCGAAACACCAAG | 60 | 91 | 99.9 |
| *PR4* | PR-4 type protein | AF061329**^1^** | | TGGTTATAGCCAGCCCCATTAG | AAGCTGCCTGTGGCAAGTG | 60 | 151 | 99.2 |
| *LOX9* | Lipoxygenase | \| NM_001281249**^1^** \| \| --- \| | | CCCTTCTTGGCATCTCCCTTA | TGTTGTGTCCAGGGTCCATTC | 60 | 101 | 90.0 |
| *PR1* | pathogenesis-related protein 1 | XM_002273752**^1^** | | GGAGTCCATTAGCACTCCTTTG | CATAATTCTGGGCGTAGGCAG | 60 | 168 | 90.0 |
| *PR2* | Class I beta-1,3-glucanase | [NM_001280967](https://www.ncbi.nlm.nih.gov/nuccore/NM_001280967)**^1^** | | TCAATGGCTGCAATGGTGC | CGGTCGATGTTGCGAGATTTA | 60 | 155 | 97.2 |
| *PR5* | thaumatin-like protein | XM_002282994**^1^** | | TTTGATCATCCTTAGGGTAGCTGTAA | ACCAATGAGTACTGTTGCAATTCC | 60 | 106 | 97.6 |
| *GST1* | glutathione S-transferase | \| NM_001281248**^1^** \| \| --- \| | | TGCATGGAGGAGGAGTTCGT | CAAGGCTATATCCCCATTTTCTTC | 60 | 98 | 90.0 |
| *PR10* | pathogenesis-related protein 10.3 | DQ396809**^1^** | | CGTTAAGGGCGGCAAAGAG | GCATCAGGGTGTGCCAAGA | 60 | 75 | 93.5 |
| *NPR1.1* | nonexpresser of *PR* genes 1 | GSVIVT00016536001**^2^** | | GACCACAACCGAGCTTCTTGATCT | ATAATCTTGGGCTCTTTCCGCATT | 60 | 108 | 93.7 |
| *PAL* | phenylalanine ammonia lyase | \| XM_003635637**^1^** \| \| --- \| | | TCCTCCCGGAAAACAGCTG | TCCTCCAAATGCCTCAAATCA | 60 | 101 | 92.9 |
| *STS* | stilbene synthase | \| NM_001281117**^1^** \| \| --- \| | | AGGAAGCAGCATTGAAGGCTC | TGCACCAGGCATTTCTACACC | 60 | 101 | 94.3 |
| *CHI* | Chalcone isomerase | NM_001281104**^1^** | | GCAGAAGCCAAAGCCATTGA | GCCGATGATGGACTCCAGTAC | 60 | 201 | 101.5 |
| *NCED2* | 9-cis-epoxycarotenoid dioxygenase 2 | NM_001281271**^1^** | | CTCTTGGCCATGTCGGAAGA | CGGAGCTGCTTGTCGAAGTC | 60 | 91 | 97.9 |

^1^ NCBI accession number, ^2^ Genoscpe Grape Genome Browser number
